# Correspondence

**Published:** 1918-03

**Authors:** Regina Flood Keyes

**Affiliations:** Hotel Royal, Paris, France


### CORRESPONDENCE

Hotel Royal, Paris, France, Nov. 18, 1917.

My dear Dr. Benedict:

Dr. Mabel Flood, U. B. 1911, and I are on our way to Serbia where we are going for the American Red Cross. Our trip across the Atlantic was most delightful and we saw no submarines.

In Bordeaux were many of our fellow countrymen in the uniform of the U. S. A., and as all soldiers or people in uniform are a part of the American Expeditionary Force, we had to go to the American Provo-marshal to report, which we did and the next day did the same thing in Paris.

From what we have seen so far, Germany will never starve the French out. They have such wonderful vegetables, and it would be better for America if we were always obliged to eat their war bread, which is dark but good. There is a shortage of butter, although we have it for breakfast, also sugar and wheat are none too plentiful.

The women run all subway trains and motor cars in many places, also street cars.

The French men who are not able to return to the front on account of their injuries, work and cover up their infirmities and go cheerfully about their affairs.

Americans are in great favor and the Red Cross much looked up to.

The Hotel Royal where we are staying has an allowance of coal, so we are not cold, although it is not overheated like an American hotel.

In Paris, the doctors are injecting the typhoid and paratyphoid all in one dose and it knocks you out fairly well for 48 hours. We had ours at Dr. Bleake's hospital. All fractures are hung up on splints which are suspended from above and joints are not immoblized.

I cannot see any difference between the old life in Paris and now except there is not so much music and every one has on some uniform. At Napoleon's Tomb today, we saw a good many airplanes and some Zeppelins which have been captured from the Germans and many, many guns, etc.

I hope every American man, woman and child will do everything that can be done to end this war. What a lesson in saving of food they could have here.

We leave on Saturday for Nice, then go to Rome and then proceed to the bottom of Italy, then to Athens, Greece, then Salonica, then nine hours by motor to Vodena, Serbia.

Very cordially,

REGINA FLOOD KEYES.

(Note: In accordance with our general policy, we have delayed publication of this letter for a month, in order to avoid any possible leak of information through hostile channels. A. L. B.)

